# Butyric and valeric glycerides alleviate sub-clinical necrotic enteritis effect on performance and gut health of broiler chickens

**DOI:** 10.1016/j.psj.2025.105441

**Published:** 2025-06-13

**Authors:** Sm Mostafizur Rahaman Sumon, Alip Kumar, Di Wu, Kosar Gharib-Naseri, Shu-Biao Wu

**Affiliations:** a School of Environmental and Rural Science, University of New England, NSW, 2351, Australia; b Perstorp Animal Nutrition, Singapore

**Keywords:** Necrotic enteritis, Organic acid, Broiler, Gene expression, Gut health

## Abstract

Organic acids are readily absorbed in the upper gastrointestinal tract, which hinders their optimal delivery to segments where gut health issues mostly occur. Glycerol esters of butyric and valeric acid (BVg) are known for their capacity to release acids within the small intestine. This study evaluated the impact of BVg on the performance and gut health of broilers under a sub-clinical necrotic enteritis (NE) challenge. A total of 1200 Cobb 500 chicks were assigned to four treatments: 1) UC- unchallenged control, 2) CC- NE challenged control, 3) BVg- CC plus BVg (1000, 500, 250 g/ton in starter, grower, and finisher phases), and 4) ANT- CC plus zinc bacitracin and salinomycin. NE challenge was induced via oral gavaging of *Eimeria* spp. vaccine (d9) and *Clostridium perfringens* (d14 and d15). Data was analyzed using JMP 16.0 employing one-way and two-way ANOVA with Tukey’s test to separate means, and Kruskal-Wallis test was used for non-normally-distributed data. During challenge period (d8-19), BVg-fed birds showed an improvement in AWG and FCR, shifting performance from CC towards UC (*P* > 0.05). Over d0-35, BVg group had similar performance with CC, however there was no significant difference observed among the BVg, ANT, and UC groups (*P >* 0.05). Male birds supplemented with BVg had a *Bifidobacterium* population nearly equivalent to those in the UC and ANT groups. The BVg supplementation showed a similar expression of *IL6* and occludin with UC and ANT groups and had a shift of expression of *IL6* from CC towards ANT group, and *MUC2* and *B^o^AT* from CC towards the UC group. Furthermore, additive supplementation shifted the oocyst counts from CC group towards the UC and ANT groups (*P >* 0.05). In conclusion, BVg has the potential to alleviate the NE-induced performance loss and gut health deterioration by modulating gut-centric gene expressions and microenvironment. Further research is warranted to explore its efficacy with varying combinations of other additives under different challenge models.

## Introduction

Necrotic enteritis **(NE)** is one of the most economically devastating enteric diseases of poultry caused by *Clostridium perfringens* **(Cp)**, which is a Gram-positive, spore-forming, anaerobic, and nonmotile rod bacterium, and normal inhabitant in the intestinal tract of poultry. The Cp population in the intestine of a healthy chicken typically ranges from 0 to 10^5^ cfu/g of digesta. However, if the count exceeds 10^6^ cfu/g of digesta, the bird becomes susceptible to infection (Si et al., 2007; Timbermont et al., 2011). The ability of the bacterium to generate a range of virulence factors, including hydrolytic enzymes and toxins, is primarily responsible for its pathogenicity (Mahamat Abdelrahim et al., 2019). The toxins produced by the Cp type A (α toxin), type C (α and β toxins) and type G (NetB toxin) are commonly associated with NE in poultry (Boulianne et al., 2020). Notably, NetB producing type G strains are thought to be the definitive cause of NE in chickens (Keyburn et al., 2008; Lee et al., 2021). The disease occurs in both clinical forms, characterized by overt clinical signs with a sudden rise in flock mortality (10–40%), and subclinical forms which do not exhibit clinical signs or mortality (Wijewanta and Seneviratna, 1971; Elwinger et al., 1992; Prescott et al., 2016; Zahoor et al., 2018; Mora et al., 2020; Abd El-Hack et al., 2022). The subclinical form is associated with higher losses in broiler production, as it often goes unnoticed by farmers. The chronic damage to the small intestinal mucosa, leads to poor performance, causing an estimated annual economic loss of US$6 billion in the global poultry industry (Wade, 2015; Moore, 2016). Historically, antibiotics have been used as therapeutic agents and then as growth promoters to prevent and control NE in the poultry industry (Beyene, 2016). However, the use of antibiotics has raised major concerns due to antimicrobial resistance **(AMR)**, posing public health risks as resistant bacteria can be transmitted from animals to humans, leading to ineffective antibiotic treatments (Hedman et al., 2020). The AMR issue and antibiotic residues in meat and eggs has led to the ban of AGPs in Europe and their voluntary phasing out worldwide (Castanon, 2007; Martin et al., 2015). These restrictions have resulted in the re-emergence of enteric diseases in poultry, including NE (Gaucher et al., 2015; Park et al., 2016). Consequently, finding suitable alternatives to antibiotics to control disease outbreaks and secure optimal production performance in the post-antibiotic era is crucial. Among the different feed additives that have shown promise as potential alternatives to antibiotics, organic acids (OAs) have gained global attention for their potential to improve gut health and growth performance (Ebeid and Al-Homidan, 2022).

Organic acids (OAs) have long been used for preserving food from microbial and fungal damage (M'Sadeq et al., 2015; Khan et al., 2022). Both the *in vitro* and *in vivo* studies have demonstrated significant benefits of using OAs, either alone or as a blend, to improve production and prevent enteric diseases in poultry over the decades. Common OAs used as feed additives in poultry production include short-chain fatty acids **(SCFAs)** like formic, acetic, propionic, butyric, and valeric acids; carboxylic acids with hydroxyl groups like lactic, malic, and citric acids; short-chain carboxylic acids with double bonds like fumaric and sorbic acids; and medium-chain fatty acids **(MCFAs)** such as caproic, caprylic, capric, and lauric acids (Adhikari et al., 2020; Scicutella et al., 2021; Ebeid and Al-Homidan, 2022; Khan et al., 2022). Although their functionality may vary due to the molecular structure of individual OAs, their positive effects are generally attributed to their antibacterial activity, modulation of gut microflora, stimulation of humoral and cell-mediated immunity, and improvement of intestinal integrity and digestibility (Gadde et al., 2017; Mora et al., 2020; Khan et al., 2022). Butyric Acid **(BA)**, a four-carbon SCFA, with its esters, salts, and coated forms, is commonly supplemented as a feed additive in the poultry industry. It is naturally produced by microbial fermentation in the large intestine and is considered a primary nutrient, providing energy to colonocytes. Additionally, BA acts as a signalling molecule that influences immunological modulation, cell differentiation, tissue development, gut-centric gene expression, and the reduction of oxidative stress (Bedford and Gong, 2018). Moreover, BA is prioritised for oxidation over other energy sources, making it a preferable energy source (Moquet et al., 2016). The inclusion of BA salts and BA glycerides in turkey diets significantly improved the feed conversion ratio (FCR), enhanced duodenal villus height, and reduced faecal populations of *Escherichia coli* and Cp (Makowski et al., 2022). The effect of free and partially protected sodium butyrate inclusion on *Salmonella enteritidis* challenged broiler chicken revealed a reduced burden of *S. enteritidis* in birds (Fernandez-Rubio et al., 2009). BA salts have also shown to regulate the immune responses by reducing serum levels of *IL-6* and *TNF-α* and by increasing serum superoxide dismutase and catalase activities in broiler chicken challenged with *Escherichia coli* lipopolysaccharide **(LPS)** (Zhang et al., 2011). Furthermore, BA reduces *Salmonella* virulence by down-regulating virulence gene expressions (Gantois et al., 2006). However, the detail mechanisms of BA related to broiler performance and gut health under NE challenge remain elusive, requiring further investigation.

Valeric acid **(VA)**, is a five-carbon fatty acid, and is relatively new feed additives in the poultry industry compared to other OAs, and much less is known about their efficacy in intestinal health and controlling enteric pathogens. A recent study investigated the effects of BA and VA glycerides separately in broiler chickens under NE challenge. It was found that both tributyrin (a butyric acid glyceride) and monovalerin (a valeric acid glyceride) improved performance and reduced the negative impact of NE, similar to bacitracin (Hofacre et al., 2020). Another study reported that VA glycerides (monovalerin) lowered the FCR in NE-challenged broilers (Onrust et al., 2018). Additionally, Gracia et al., (2024) showed that a combination of BA, VA glycerides, and oregano oil improved gut health and performance in commercial broilers. However, the inconsistency in performance at different VA inclusion rates and the limited understanding of its interactions with gut health requires further investigation. While BAs effects are well-documented, VA is comparatively new, and their combined impact on alleviating NE and promoting gut health remains unexplored. It is hypothesized that combining butyric and valeric acid glycerides may have synergistic effects and may promote healthy gut microbiota, trigger immune responses, and enhance nutrient transport and gut barrier functions under diseased conditions, ultimately improving performance. Therefore, this study aimed to evaluate the combined effects of butyric and valeric acids glycerides **(BVg)** on growth performance and gut health, and to elucidate their underlying mechanisms in broiler chickens challenged with subclinical NE.

## Material and methods

### Animal ethics

The experimental procedures applied in the current study were approved by the Animal Ethics Committee of University of New England, Armidale, NSW 2351, Australia (ARA22-047). The Australian Bureau of Animal Health-accredited criteria for the care and use of laboratory animals for research purposes were adhered to during the experiment.

### Birds and husbandry

A total of 1200 d-old chicks (Cobb 500) were obtained from Baiada Hatchery in Tamworth, NSW. On arrival, chicks were vent sexed and allocated to 40 pens, each stocked with 30 birds (15 males and 15 females) in the closed and power-ventilated poultry shed at Rob Cumming Poultry Research Station, Kirby, University of New England, NSW. Birds were raised in a climate-controlled house with softwood shavings as bedding materials to a depth of 8 cm. Each pen featured two feeders and four nipple drinkers providing *ad libitum* feed and fresh water. Lighting, temperature and relative humidity were maintained following Cobb 500 guidelines.

### Experimental design and dietary treatments

The experiment was conducted using a randomized design with 4 treatments, each replicated 10 times, with 30 birds per replication. The treatments and experimental design are shown in Table 1. Diets were based on wheat, soybean meal and sorghum, and were fed in three phases: starter (d0-8), grower (d8-19), and finisher (d19-35). The additives included a blend of butyric and valeric glyceride (Gastrivix^TM^ Avi, Perstorp Waspik BV, Netherlands) at l000, 500 and 250 g/ton in the respective phases. Diets were formulated to meet the nutrient requirements of Cobb 500 birds, considering nutrients and the matrix values of phytase (Quantum Blue 5000 G) at 500 FTU/kg. Prior to feed formulation, the nutrient contents of feed ingredients were determined using near-infrared spectroscopy (AminoNIR®, Evonik AminoProx, Essen, Germany). Crumbled diets were fed in the starter phase, whereas cold pelleted diets were used in the grower and finisher phases. The feed additive and antibiotic were added to the feed mix before pelleting. Detailed diet compositions for each phase are shown in Table 2.

**Table 1:** Experimental design and treatments.

Treatment	Diet	Additive/Antibiotic, %		
		Starter d0-8	Grower d8-19	Finisher d19-35
UC- Unchallenged Control	Control diet: Wheat-SBM-Sorghum	0	0	0
CC- Challenged Control	Control diet + NE challenge	0	0	0
BVg- Blend of butyric and valeric glyceride	Control diet + NE challenge + BVg	0.100	0.050	0.025
ANT- Antibiotic Control	Control diet + NE challenge + ANT	Zn bacitracin: 0.0267 + Salinomycin: 0.050	Zn bacitracin: 0.0267 + Salinomycin: 0.050	Zn bacitracin: 0.0267 + Salinomycin: 0.050

NE: Necrotic enteritis

**Table 2:** Diet composition and nutrient contents.

Ingredients (as-fed basis, %)	Starter (d0-8)	Grower (d8-19)	Finisher (d19-35)
Wheat	50.9	56.1	61.9
SBM	33.0	27.7	22.5
Sorghum	10.0	10.0	10.0
Canola oil	2.33	2.45	2.62
Dicalcium Phosphate	1.15	0.420	0.272
Limestone	1.11	1.14	1.09
L-lysine HCl 78.4%	0.344	0.350	0.368
DL-methionine	0.330	0.305	0.280
Salt	0.223	0.220	0.230
L-threonine	0.180	0.180	0.180
UNE Vit concentrations^1^	0.075	0.075	0.075
UNE TM concentrations^2^	0.075	0.075	0.075
Choline Chloride 60%	0.045	0.079	0.075
Na bicarb	0.017	0.039	0.026
Phytase^3^	0.010	0.010	0.010
Titanium dioxide	0	0.500	0
Sand^4^	0.200	0.403	0.200
**Calculated nutrients**^5^			
Dry Matter, %	89.6	89.6	89.4
AME, kcal/kg	2,950	3,000	3,080
Crude Protein, %	22.5	20.5	18.7
Crude fat, %	3.65	3.79	3.98
Crude Fiber, %	2.78	2.67	2.59
Digestible Arginine, %	1.31	1.16	1.03
Digestible Lysine, %	1.28	1.16	1.06
Digestible Methionine, %	0.609	0.562	0.519
Digestible Met + Cys, %	0.941	0.876	0.816
Digestible Tryptophan, %	0.288	0.263	0.240
Digestible Isoleucine, %	0.843	0.753	0.670
Digestible Threonine, %	0.869	0.796	0.729
Digestible Valine, %	0.887	0.803	0.727
Calcium, %	0.960	0.800	0.740
Available phosphorus, %	0.540	0.400	0.370
Sodium, %	0.170	0.170	0.170
Potassium, %	1.00	0.905	0.819
Chloride, %	0.25	0.254	0.265
Choline, mg/kg	1,732	1,793	1,700
Linoleic acid (18:2), %	1.27	1.31	1.37

AME = apparent metabolizable energy.

1 Vitamin premix provided the following per kilogram diet: vitamin A, 12,000,000 IU; vitamin D, 5,000,000 IU; vitamin E, 75 mg; vitamin K, 3 mg; cyanocobalamin,0.016 mg; folic acid, 2 mg; riboflavin, 8 mg; pyridoxine, 5 mg; biotin, 0.25 mg; thiamine, 3 mg; nicotinic acid, 55 mg; pantothenic acid, 13 mg and antioxidant ethoxyquin,50 mg.

2 Mineral premix provided the following per kilogram diet: Cu sulfate, 16 mg; Mn sulfate, 60 mg; Mn oxide, 60 mg; I (iodide), 0.125 mg; Se (selenite), 0.3 mg; Fe sulfate,40 mg; Zn oxide and sulfate, 100 mg.

3 Phytase: Quantum Blue 5G (100 g/t)

4 Sand was replaced with the required amount of BVg and added to the top.

5 Nutrient contents were measured using near-infrared spectroscopy (AminoNIR®, Evonik AminoProx, Germany).

### Proximate analysis of diet

Representative feed samples (∼ 200 g) were collected from various replicates of each treatment across all phases (starter, grower, and finisher) during feed distribution and stored at 4°C until analysis. Prior to analysis, the samples were ground in a laboratory mill equipped with a 1-mm die. Proximate analysis was conducted in duplicate, following AOAC International methods (Horwitz and Latimer, 2000). Dry matter content was determined by drying samples (∼ 2-5 g) at 105 °C for 16 to 24 hours using a hot air oven. The gross energy content of the feed was measured using an oxygen bomb calorimeter (Parr™ 6400 Automatic Isoperibol Calorimeter, Parr Instrument Company, Moline, Illinois, USA), using 0.8 g to 1.10 g of sample as per the manufacturer's manual. Nitrogen content was assessed via the Kjeldahl method (Kelplus Classic, DX VA, Pelican Equipment) and crude protein was estimated by multiplying nitrogen percentage by 6.25. Crude fat was determined by the solvent extraction method using Soxtec^TM^ 8000 extraction system (Foss, Denmark) with diethyl ether (boiling point, 40–60 °C) as the solvent. The crude fibre was determined by digesting the moisture and fat-free sample with acid and base solution using ANKOM^2000^ Fibre Analyzer (Ankom Technology, Macedon, New York, USA) following the manufacturer’s instructions. Table 3 presents the major nutrient composition of experimental diets.

**Table 3:** Analysed major nutrient composition of diet in different feeding phases.

Components	UC^1^	CC	CC + BVg	CC + ANT
Starter (d0-8***)***				
Dry matter (%)	88.7	88.8	89.0	89.2
Gross energy (Kcal/kg)	3916	3914	3921	3921
CP (%)	21.9	22.2	22.2	21.9
Crude fat (%)	4.85	4.34	4.20	4.17
Crude fibre (%)	2.00	3.06	3.60	2.55
Grower (d8-19)				
Dry matter (%)	88.8	88.8	88.1	89.1
Gross energy (Kcal/kg)	3904	3918	3925	3920
CP (%)	19.5	19.7	19.8	19.8
Crude fat (%)	5.43	4.26	4.30	4.42
Crude fibre (%)	2.79	2.85	3.21	3.22
Finisher (d19-35)				
Dry matter (%)	87.6	87.7	87.2	87.0
Gross energy (Kcal/kg)	3930	3946	3943	3944
CP (%)	17.9	17.9	17.9	18.1
Crude fat (%)	4.51	4.34	4.51	4.62
Crude fibre (%)	2.92	1.83	2.92	3.32

1 UC: Unchallenged control; CC: Challenged control; BVg: Blend of butyric and valeric glyceride @ 1000 g/t, 500 g/t, 250 g/t in starter, grower, and finisher phases, respectively; ANT: Antibiotic, Zn bacitracin 267 g/t; Salinomycin 500 g/t

### Necrotic enteritis challenge

Challenged birds were given 1 mL/bird *per os Eimeria spp* vaccine strains containing *E. acervulina* 5,000, *E. maxima* 5,000 and *E. brunetti* 2,500 oocysts on d9. The unchallenged control groups were orally gavaged with PBS. On d14 and d15, birds in the challenged groups received 1 mL/bird *per os Clostridium perfringens* EHE-NE18 (approximately 10^8^ cfu/mL), while unchallenged birds received PBS as a sham treatment (Wu et al., 2010; Rodgers et al., 2015).

### Evaluation of growth performance

Pen weight and feed intake were measured on arrival (d0) and on days 8, 19, 28 and 35. These measurements were used to calculate average bird weight gain **(AWG)**, average feed intake **(AFI)**, and feed conversion ratio **(FCR)**. The FCR was corrected for mortality by adding the weight of dead chickens back to the pen weight within each respective period. European production efficiency factor **(EPEF)** was determined at d35 using the following formula,

$$EPEF=\;\frac{Liveweight\;\left(kg\right)\;at\;d35\;\times \;Livability\;at\;d35\;\left(\%\right)}{Age\;in\;days\;\times FCR\;\left(d0-35\right)}\;\times \;100$$

### Sampling and NE lesion scoring

On d16, jejunal section, caecal content and ileal digesta were collected from randomly selected four birds (2 male and 2 female) of each pen. Birds were electrically stunned and subsequently euthanized by cervical dislocation, and dissection was performed. After dissection, the entire length of the small intestine was removed to examine the NE lesions. The intestinal lesion score was determined by 2 experienced researchers with no knowledge of the treatment allocation of the birds using a scoring system ranging from 0 to 6 (Keyburn et al., 2006; Keyburn et al., 2008). Approximately 2 cm of the proximal jejunum tissue was excised, flushed with PBS (4°C) and collected in 2 mL Eppendorf tubes filled with RNAlater (Qiagen, Germany). These tissue sections were kept at 4°C for 24 h, and then stored at -20°C until RNA extraction. Pooled male (2 male) and pooled female (2 female) caecal content (as much as possible) was aseptically collected into sterile 60 mL container, mixed thoroughly, and transferred approximately 1 g to a 2 mL Eppendorf tube. The tubes were snap-frozen in liquid nitrogen and stored at -20°C until DNA extraction. The remaining caecal contents were stored in the freezer (-20°C) until short-chain fatty acid (SCFA) determination. Similarly, ileal digesta was collected using the same procedure and stored at -20°C until SCFA analysis.

On d 18, pooled freshly devoided excreta droppings (at least 5 droppings from each pen) were collected in a 40 mL plastic container, thoroughly mixed, and a small quantity of excreta was transferred to 2 mL Eppendorf tubes and stored at 4°C for *Eimeria* spp. oocyst counting. The oocysts were enumerated within 7 days of sample collection.

### Eimeria oocyst count

Excreta samples (100 mg each) were diluted with 900 mL of saturated salt solution (relative density, 1.3). After mixing by vortexing, the samples were refrigerated at 4°C for 1 hour to facilitate the flotation of oocysts and settling of sample debris. Then, 600 μL saturated salt solution was added to the Whitlock chamber (Whitlock universal slides, JA Whitlock & Co., NSW 2122, Australia) and 150 μL of diluted samples were pipetted from the top layer of the samples into the Whitlock chamber. Oocysts were counted using a 10 × objectives and the data were expressed as oocyst/gram, with the counts in the chamber multiplied by 100 as the dilution factor.

### SCFA Determination

A previously described technique, with minor modifications, was employed to analyse the caecal and ileal SCFA (Jensen et al., 1995). In short, approximately 0.8 g of caecal and 1.5 g of ileal digesta were weighed into centrifuge tubes and placed on ice and mixed with 1 mL of an internal standard (0.01M ethyl butyric acid). The mixture was vortexed and centrifuged at 15,000 RCF for 20 min at 5°C. Around 1 mL of supernatant was carefully transferred into 8 mL vials that had been set out on ice. Then, 0.5 mL of concentrated HCl (36%) and 2.5 mL of diethyl ether were added to the solution and vortexed for 1 min followed by centrifugation for 15 min at 3000 RCF at 5°C. After centrifugation, 400 µL supernatant was transferred into 2 mL gas chromatograph **(GC)** vials in duplicate, and then 40 µL N-tert-butyldimethylsilyl-N-methyl trifluoroacetamide **(MTBSTFA)** was added to each vial. After gentle vortexing, samples were heated at 80°C for 20 min in a heating block. The GC vials were securely tightened and left at room temperature for 48 h prior to analysis. Analysis was performed using a gas chromatograph (Varian CP3400 CX, Varian Analytical Instruments, Palo Alto, CA, USA). The concentrations of caecal and ileal SCFA were expressed as µmol/g digesta.

### Determination of Fluorescein Isothiocyanate-dextran (FITC-d) Concentration

All the birds to be sampled on d16 were gavaged with 1 mL fluorescein isothiocyanate-dextran (**FITC-d**) at a dosage of 4.17 mg/kg of average body weight (molecular weight 4000, Sigma–Aldrich Co., Sydney, Australia) around 2.5 h prior to euthanization. Birds were electrically stunned, and blood samples were collected from the jugular vein in clot activator vacutainer tubes. Blood samples were left at room temperature for about 3 h, and then centrifuged at 1,500 RCF for 10 min to collect serum, which was stored at -20°C until FITC-d analysis. Fluorescent levels in serum were measured with an excitation wavelength of 485 nm and an emission wavelength of 528 nm on a Synergy HT, Multimode microplate reader (SpectraMax M2e, Molecular Devices, CA) (Kuttappan et al., 2015). The fluorescence levels were converted to FITC-d concentration (µg/mL) based on a calculated standard curve obtained from known levels of FITC-d.

### DNA Extraction

The DNeasy PoweSoil Pro Kit (Qiagen, Inc., Hilden, Germany) was used, with minor adjustments to extract the DNA from frozen caecal samples that were obtained on d16. Approximately 100 mg of caecal content and 300 mg of glass beads (0.1 mm) were added to a 2 mL Eppendorf tube. Then 700 µL of CD1 solution was added and mixed by quick vortexing. Later, the tubes were placed in Tissuelyser II for 5 min at a frequency of 30 times per second to disrupt bacterial cells. The samples were then incubated at 90°C for 10 min in a heating block followed by centrifugation at 15,000 RCF for 1 min. About 500 µL supernatant was transferred into a new 2 mL Eppendorf tube with proper labelling and 250 μl of CD2 solution was added to each tube and vortexed. After that, samples were centrifuged at 4500 RCF for 5 min and supernatants were transferred to a new S-block. The S-block was placed inside of QIAcube HT robotic machine (Qiagen, Inc., Hilden, Germany) and the extraction was performed according to the manufacturer’s instructions. DNA concentration and purity were evaluated using a NanoDrop ND-8000 spectrophotometer (Thermo Fisher Scientific, Waltham, MA). DNA samples with a 260/280 purity ratio over 1.8 were deemed suitable and stored at -20°C for subsequent analysis.

### Caecal Bacterial Load Analysis

The caecal bacterial DNA load was determined following a previously published protocol (Kheravii et al., 2017). In brief, the stored caecal DNA was thawed and diluted 20-fold in nuclease-free water. Quantification of the 8 major bacterial groups was conducted using a quantitative real-time PCR (RT-qPCR) system, using the Rotorgene 6000 (Qiagen, Inc., Hilden, Germany). Each reaction had a total volume of 10 µL, consisting of 2 µL of diluted caecal DNA, 0.4 µL (300 mmol/L) of each forward and reverse primer, 2.2 µL of nuclease free water (NFW) and 5 µL of 2 × master mix containing SYBR-Green. The SYBR-green Mix (SensiMix SYBR No-Rox, Bioline, TN, USA) was used for the genomic DNA copies of *Lactobacillus* spp., *Bifidobacterium* spp., *Bacteroides* spp., *Bacillus* spp., *Ruminococcus* spp., Enterobacteriaceae, and total anaerobic bacteria. The specific 16S rRNA primers applied for quantification of these bacterial groups are detailed in supplementary Table S1. The number of target DNA copies was calculated, and bacterial quantity was expressed as log_10_ (genomic DNA copy number)/g digesta.

### RNA Extraction and cDNA Synthesis

Total RNA was extracted from each jejunal sample using the RNeasy Mini Kit (Qiagen, Hilden, Germany) following the manufacturer’s instructions. The extracted RNA was quantified with a NanoDrop ND-8000 spectrophotometer (Thermo Fisher Scientific, Waltham, MA) and assessed for integrity using the Agilent TapeStation-4200 (Agilent Technologies, Inc., Waldron, Germany). The RNA samples with a ratio of 260/230 greater than 2.0, 260/280 between 2.0 to 2.2, and a RIN of more than 7 were considered high quality. The concentration of extracted RNA was adjusted to 100 ng/µL before cDNA synthesis. The SensiFast cDNA synthesis kit (Meridian Bioscience, Sydney, NSW, Australia) was used to reverse transcribe the extracted RNA into cDNA using the real-time PCR machine (Rotor-Gene Q, QIAGEN GmbH, Hilden, Germany). The obtained cDNA was diluted 10 times with NFW and stored at -20°C for further analysis.

### Real-Time Quantitative PCR

The qPCR analysis was performed with cDNA template in duplicates using the SensiFAST SYBR No-ROX kit (Meridian Bioscience, Sydney, NSW, Australia) with a real-time PCR machine (Rotor-Gene Q, QIAGEN GmbH, Hilden, Germany). Primers targeting specific genes of interest related to gut integrity, immunity, nutrient transporters, and cell apoptosis are listed in supplementary Table S2. The PCR reaction was carried out in a volume of 10 μL containing 5 μL of 2 × SensiFAST, 0.4 μL of each primer, 2.2 μL of NFW and 2 μL of 10 × diluted cDNA template. After the cycle, 2 stable reference genes with the lowest M-value (<0.5), *ACTB* (M-value = 0.338) and *RPL4* (M-value = 0.338) were chosen by calculating the gene expression stability measurement (geNorm M) of eight widely used house-keeping genes in qbase+ software version 3.0 (Biogazelle, Zwijnbeke, Belgium). These genes were: ribosomal protein L4 ***(RPL4)****,* TATA-Box Binding Protein ***(TBP)****,* glyceraldehyde-3-Phosphate *dehydrogenase* ***(GADPH)****,* hydroxymethylbilane synthase ***(HMBS)****,* tyrosine 3- monooxygenase/tryptophan 5-monooxygenase activation protein zeta ***(YWHAZ)****,* succinate dehydrogenase subunit A ***(SDH A)****,* beta actin ***(ACTB)*** and *18s rRNA* genes. The amplification cycle (Cq) values for candidate target genes were obtained and imported into qBase+ version 3.0 software (Biogazelle, Zwijnbeke, Belgium), which were then analysed against the reference genes. qBase+ employed the geometric mean method to convert logarithmic Cq values to a linear relative quantity using the exponential function for relative gene quantification. The resulting data were then exported for statistical analysis. For every target gene, the normalised relative quantities (NRQ) values were computed and analysed for all samples.

### Data Analysis

All the data generated in this study were tested for normal distribution before statistical analysis. The performance data were analysed as a completely randomised design using JMP 16.0 (SAS Institute, Cary, NC, USA) where the pen was served as an experimental unit (n = 40). Since the challenge was introduced on d9, birds in the UC and CC pens were considered as a single group (Control) until d8 (the end of the starter phase). The data were analysed for the treatment effect with female percentage (corrected to dead birds) as a covariate when it was significant. The significant differences between means were separated by Tukey’s test. To determine the main effects of the experimental treatment and sex, and their interaction, the measurements (serum FITC-d level and bacterial loads) obtained from both male and female birds were subjected to a 2-way ANOVA analysis according to a 4 × 2 factorial design. The means were considered significantly different when *P*-value was <0.05 and declared a tendency to be different with 0.05 < *P* < 0.10. Additionally, outcomes were referred to as ‘shifting’ when they show a transitional or intermediate response between treatment groups, where the result was not significantly different from either group but indicating a progression toward recovery (control group). The *Eimeria* oocyst counts, intestinal lesion scores and gene expression data were analysed using the non-parametric Kruskal-Wallis test as the data were not normally distributed.

## Results

### Growth performance

**Fig. 1:**
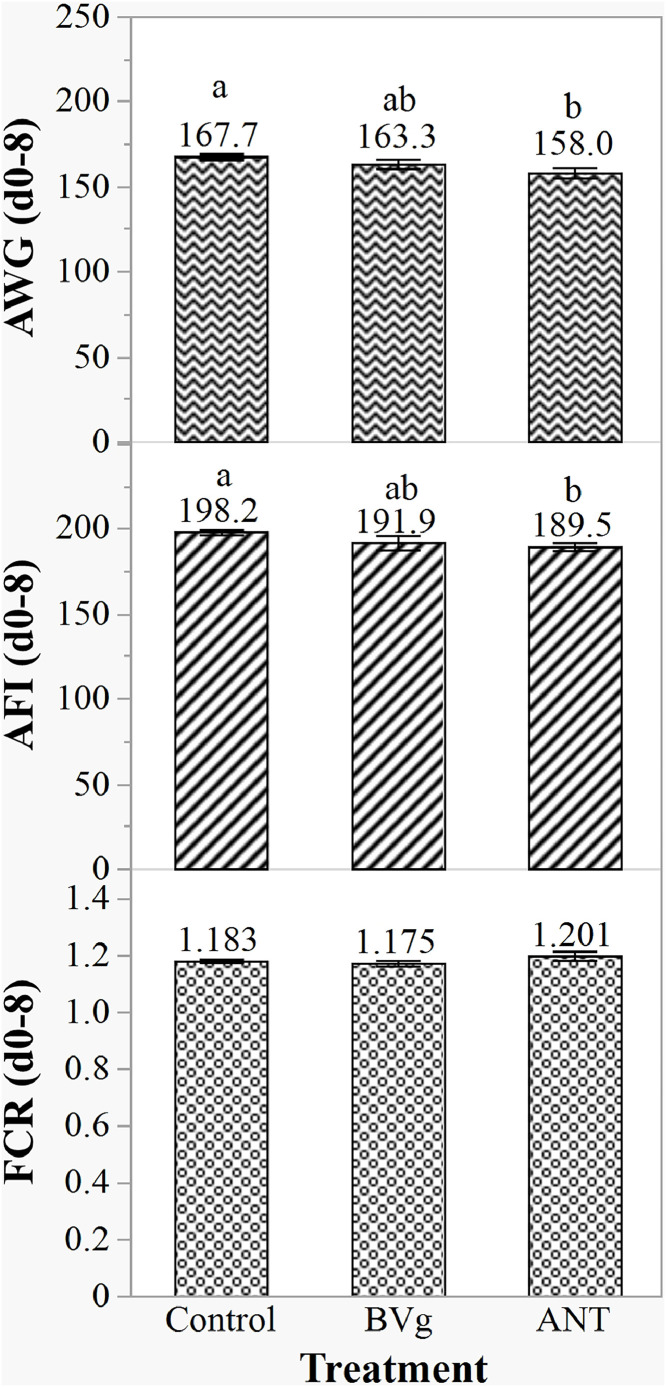
Effect of BVg on growth performance of broilers before inducing NE challenge (d 0–8). AFI and AWG were recorded and expressed in g. Control: unchallenged birds fed basic diet; BVg: Blend of butyric and valeric glyceride @ 1000 g/t, 500 g/t, 250 g/t; ANT: Antibiotic, Zn bacitracin 267 g/t; Salinomycin 500 g/t. ^a-b^Values within a column with different letters differ significantly (*P* < 0.05).

At the starter phase and before inducing challenge (d 0-8), control group had significantly (*P* < 0.05) higher AWG and AFI compared to the ANT groups, while BVg was intermediate between control and ANT groups. No difference of FCR was observed among the treatments (Fig. 1).

During the grower phase (d 8-19), when the NE challenge was induced, birds in the CC group had a significantly lower AWG and a higher FCR compared to the UC group (*P* < 0.001), indicating a successful NE challenge (Table 4). Birds fed ANT showed significantly higher AWG and lower FCR (*P* < 0.001) than the CC group. The AWG and FCR in the BVg group did not significantly differ from both the CC or UC groups during this period (*P* > 0.05) indicating the BVg supplementation shifted the AWG and FCR from CC group towards the UC group during this period. During the finisher phase (d 19–35), the AWG, AFI, FCR, and liveability were not significantly affected by the NE challenge, additive, or ANT supplementation (*P* > 0.05).

**Table 4:** Effect of BVg on growth performance of broilers under necrotic enteritis challenge.

Treatment^1^	Grower Phase (d8-19)				Finisher Phase (d19-35)				Overall Study (d0-35)				EPEF
	AWG (g)	AFI (g)	FCR	Liveability, %	AWG (g)	AFI (g)	FCR	Livaebility, %	AWG (g)	AFI (g)	FCR	Liveability, %	
UC	655^ab^	906	1.384^bc^	98	1577	2655	1.686	98	2402^ab^	3723	1.551^b^	93	420^ab^
CC	608^c^	885	1.455^a^	98	1535	2663	1.736	99	2308^b^	3709	1.607^a^	94	393^b^
CC + BVg	616^bc^	872	1.416^ab^	99	1572	2685	1.707	99	2353^ab^	3718	1.580^ab^	94	409^ab^
CC + ANT	663^a^	911	1.375^c^	98	1630	2689	1.651	98	2450^a^	3757	1.533^b^	93	434^a^
SEM^2^	10	12	0.010	0.7	23	44	0.022	0.9	25	48	0.013	2	10
*P*-value	<0.001	0.115	<0.001	0.705	0.058	0.937	0.082	0.546	0.005	0.906	0.003	0.970	<0.034

AWG: average weight gain; AFI: average feed intake; FCR: feed conversion ratio; EPEF: European production efficiency factor

^a-c^Values within a column with different letters differ significantly (*P* < 0.05).

1 Treatment abbreviations: UC: Unchallenged control; CC: Challenged control; BVg: Blend of butyric and valeric glyceride @ 1000 g/t, 500 g/t, 250 g/t in starter, grower, and finisher phases, respectively; ANT: Antibiotic, Zn bacitracin 267 g/t; Salinomycin 500 g/t.

2 SEM: standard error of mean.

Considering the course of entire trial (d0-35), birds in the CC groups exhibited a higher FCR compared to those in the UC groups (*P* < 0.05) (Table 6). Birds supplemented with ANT had a significantly (*P* < 0.001) higher AWG and a lower FCR in comparison with the CC group, albeit they were not different from the UC group. The BVg supplemented birds did not show significantly different AWG and FCR from those in the CC, UC and ANT groups (*P* > 0.05), indicating a shift of these two performance parameters from the CC group towards the ANT and UC groups, confirming the observations made during d 8-19. Birds receiving ANT had significantly higher EPEF compared to the CC group and those of BVg and UC were intermediate indicating BVg treatment shifted EPEF from CC towards UC and ANT groups.

### Intestinal Lesions and Gut Permeability

The presence of lesions in the different segments of intestine of all sampled birds is shown in supplementary Table S3. The results indicated that the experimental treatments had no significant effect on intestinal lesions (*P* > 0.05) which indicates mild subclinical NE challenge.

**Fig. 2:**
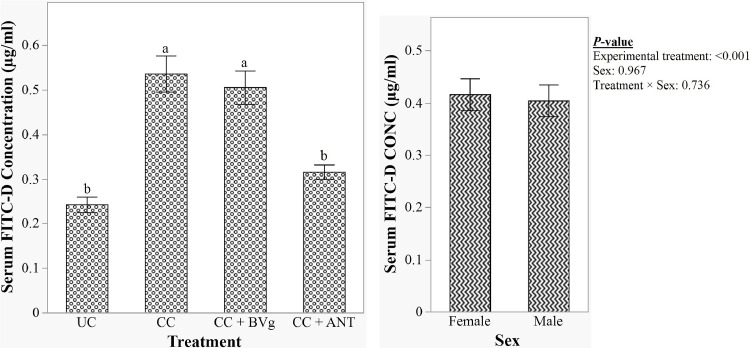
Effect of BVg on gut permeability of broilers under necrotic enteritis challenge on d16. Treatment abbreviations: BVg: Blend of butyric and valeric glyceride @ 1000 g/t, 500 g/t, 250 g/t in starter, grower, and finisher phases, respectively; ANT: Antibiotic, Zn bacitracin 267 g/t and Salinomycin 500 g/t. ^a-b^Values within a column with different letters differ significantly.

As shown in Fig. 2, NE challenge significantly increased serum FITC-d concentration in the CC group compared to the UC birds (*P* < 0.001) further confirmed NE challenge effect on birds. The ANT supplementation significantly reduced (*P* < 0.001) intestinal permeability compared to the CC group, but showed no significant difference from the UC group. BVg supplementation did not affect FITC-d concentration compared to the CC group (*P* > 0.05). Neither the sex as a main effect (*P* = 0.967) nor the sex and treatment interaction (*P* = 0.736) on intestinal permeability was observed.

### Effect on Eimeria Oocyst

**Fig. 3:**
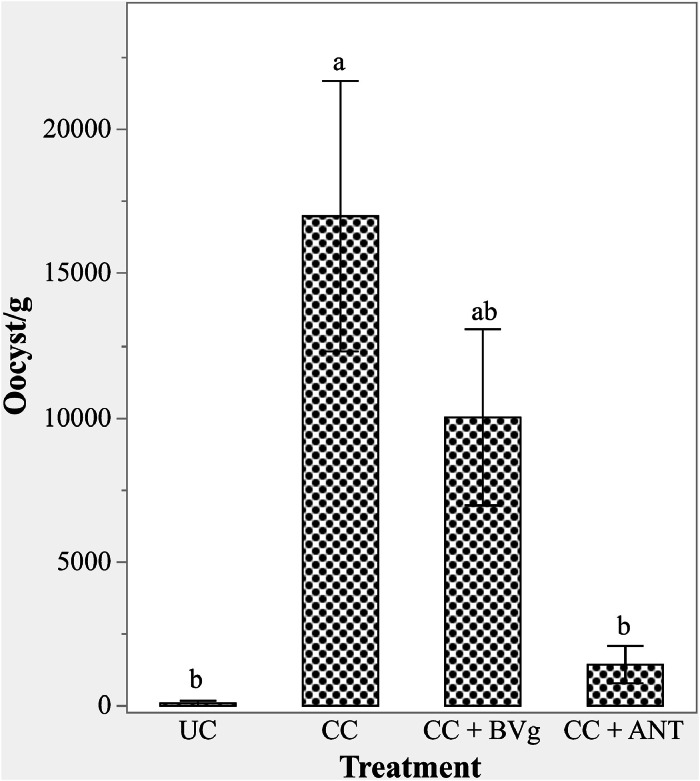
Effect of BVg on total excreta oocyst counts of broilers under necrotic enteritis challenge on d18 . Treatment abbreviations: UC: Unchallenged control; CC: Challenged control; BVg: Blend of butyric and valeric glyceride @ 1000 g/t, 500 g/t, 250 g/t in starter, grower, and finisher phases, respectively; ANT: Antibiotic, Zn bacitracin 267 g/t; Salinomycin 500 g/t. ^a-b^Values within a column with different letters differ significantly (*P* < 0.001).

The excreta mean oocyst counts are shown in Fig. 3. Compared to the UC groups, the CC groups exhibited significantly higher oocyst count (*P* < 0.001). Birds receiving ANT supplementation had lower number of oocysts compared to the CC groups (*P* < 0.001), but not statistically different from the birds in the UC groups (*P* > 0.05). The birds fed BVg did not differ significantly from CC, UC and ANT groups, indicating a shift of the oocyst count towards the levels observed in the UC and ANT groups.

### Caecal and Ileal SCFAs

The effects of NE challenge and additive supplementation on caecal and ileal SCFA in broilers on day 16 are shown in supplementary Table S4 and Table 5. There was a tendency towards the differences among the treatments on the propionate (*P* = 0.071) and isovalerate (*P* = 0.075) levels in the caecum, with BVg supplemented birds tended to have higher concentrations of both propionate and isovalerate compared to the UC group. Significant difference was observed in the ileal lactic acid concentrations (*P* < 0.001) among the treatments, where birds in the CC group had significantly higher ileal lactate level compared to the UC groups (*P* < 0.05). Antibiotic supplementation significantly reduced lactate level compared to the CC group (*P* < 0.05) but was not different from UC group (*P* > 0.05). Ileal lactic acid concentrations in birds fed BVg was not different from birds in the CC group (*P* > 0.05) and was elevated compared to the UC and ANT groups (*P* < 0.05).

**Table 5:** Effects of BVg on the SCFA concentrations (μmol/g) in ileal digesta of broilers.

Treatments^1^	SCFA (µmol/g)				
	Formic acid	Acetic acid	Propionic acid	Lactate	Succinate
UC	0.28	2.91	0.12	4.89^b^	0.13
CC	0.22	2.48	0.15	14.6^a^	0.16
CC + BVg	0.28	1.65	0.25	19.0^a^	0.12
CC + ANT	0.45	2.92	0.16	3.91^b^	0.08
SEM^2^	0.12	0.39	0.07	2.45	0.05
*P*-value	0.551	0.101	0.606	<0.001	0.657

SCFA: short chain fatty acids

^a-b^Values within a column with different letters differ significantly (*P* < 0.05).

1 Treatment abbreviations: UC: Unchallenged control; CC: Challenged control; BVg: Blend of butyric and valeric glyceride @ 1000 g/t, 500 g/t, 250 g/t in starter, grower, and finisher phases, respectively; ANT: Antibiotic, Zn bacitracin 267 g/t; Salinomycin 500 g/t.

2 SEM: standard error of means.

### Bacterial Quantification of Caecal Digesta

**Fig. 4:**
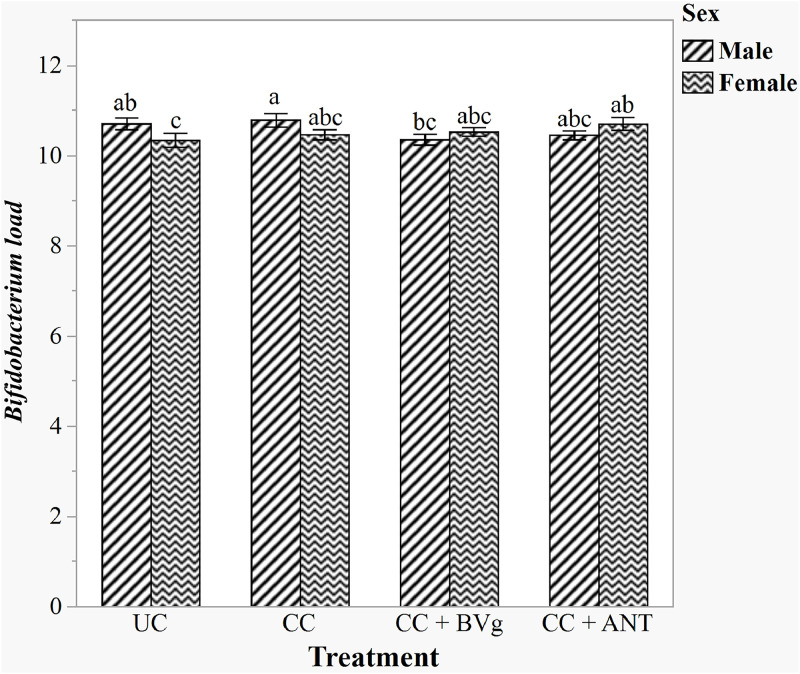
Interaction of experimental treatment and sex on caecal *Bifidobacterium* spp. loads on d 16. Treatments: BVg: Blend of butyric and valeric glyceride @ 1000 g/t, 500 g/t, 250 g/t in starter, grower, and finisher phases, respectively; ANT: Antibiotic, Zn bacitracin 267 g/t; Salinomycin 500 g/t. ^a-c^Mean values with different superscripts differ significantly (*P* < 0.05).

The main effects of experimental treatment and sex, and their interaction on the caecal microbial population on day 16 are illustrated in Table 6. There were no interactions between dietary treatments and sex across all the bacterial loads analysed except *Bifidobacterium* (*P* > 0.05). Therefore, only main effects are described below. For the effect of dietary treatments, birds in the CC and BVg groups had significantly higher *Bacteroides* load (*P* < 0.05) than the UC and ANT groups, and supplementation with BVg did not show effect on the *Bacteroides* load compared to the CC groups. *Lactobacillus, Bacillus* and the total bacterial loads showed significant changes in response to sex as a main effect. Female birds had higher *Lactobacillus* spp. (*P* = 0.021), *Bacillus* spp. (*P* < 0.001), and total bacterial loads (*P* = 0.012) compared to males. Fig. 4 illustrates the interaction between sex and experimental treatments on *Bifidobacterium* spp. in caeca. Supplementation of BVg significantly reduced *Bifidobacterium* loads compared to the CC group only in male birds (*P* < 0.05) and were not different from UC and ANT supplemented groups (*P* < 0.05). In female birds, on the other hand, BVg supplementation had similar effect on *Bifidobacterium* population with all the other treatment groups while ANT supplementation increased *Bifidobacterium* loads compared to the UC groups.

**Table 6:** Experimental treatment and sex as main effects on caecal microbiota load on d 16.

Parameters	Concentration of caecal microbiota (log10 copies/g digesta)					
	*Lactobacillus*	*Ruminococcus*	*Bacteroides*	*Bacillus*	*Enterobacter*	Total
Experimental treatment						
UC	9.14	10.8	10.2^b^	9.05	9.34	11.8
CC	9.21	10.8	10.6^a^	8.88	9.26	11.8
CC + BVg	9.19	10.8	10.6^a^	9.00	9.19	11.9
CC + ANT	9.03	9.8	10.2^b^	9.30	9.09	11.7
SEM	0.08	0.4	0.1	0.12	0.13	0.1
Sex						
Female	9.24^a^	10.3	10.5	9.28^a^	9.27	11.9^a^
Male	9.05^b^	10.8	10.3	8.84^b^	9.18	11.7^b^
SEM	0.06	0.3	0.1	0.09	0.09	0.1
*P*-value						
Experimental treatment	0.384	0.191	0.002	0.108	0.582	0.105
Sex	0.021	0.146	0.060	<0.001	0.503	0.012
Treatment × Sex	0.904	0.131	0.441	0.715	0.402	0.854

^a-b^Values within a column with different letters differ significantly (*P* < 0.05).

^1^Treatment abbreviations: UC: Unchallenged control; CC: Challenged control; BVg: Blend of butyric and valeric glyceride @ 1000 g/t, 500 g/t, 250 g/t in starter, grower, and finisher phases, respectively; ANT: Antibiotic, Zn bacitracin 267 g/t; Salinomycin 500 g/t.

^2^SEM: standard error of means.

### Expression of Gut Integrity and Immunity Related Genes

As the data were not normally distributed, nonparametric analyses were performed and thus no interactions were able to be detected. Therefore, we present the paired comparison firstly focusing on within each sex to examine whether the dietary treatments show same or different effects on male and female birds, and then examine the sex effect within each dietary treatment as shown in Table 7. The dietary treatment had significant effects on the expression of *IFN-y* and *IgG* only in male birds, and *IL6* only in female birds (*P* < 0.05). *IFN-y* was upregulated in the male birds of the CC and BVg groups compared to the UC group (*P* < 0.05), while *IgG* was downregulated in male birds of all the three challenged groups compared to UC group (*P* < 0.05). The expression *IFN-y* and *IgG* in BVg groups in both the male and female birds were not different from CC and ANT groups (*P* > 0.05). In contrast, *IL6* was upregulated in the female birds of CC groups compared to ANT groups (*P* < 0.05). However, expression of *IL6* in BVg supplementation in both the male and female birds were not different from UC and ANT groups, showing a shift from CC towards ANT group in the female birds (*P* > 0.05). No differences were observed for other genes. Furthermore, sex affected the expression of *TJP1, OCLN* and *IgG* (*P* < 0.05). The expression of *TJP1* was upregulated in female birds compared to male birds in the dietary treatment groups UC, BVg and ANT (*P* < 0.05) but not in CC (*P* > 0.05), and *OCLN* in the group BVg (*P* < 0.05) but not in other three groups (*P* > 0.05). However, *IgG* was upregulated in male birds compared to female birds in the UC group (*P* < 0.05) but not in other groups (*P* > 0.05).

**Table 7:** Effects of experimental treatment and sex on jejunal expression of gut integrity and immunity related genes on d 16.

Sex	Experimental Treatment	Tight junction proteins			Immunity related genes					
		TJP1	OCLN	JAM2	*IFN-y*	*IgA*	*IgG*	*IgM*	*IL2*	*IL6*
Female	UC	1.32^a^	1.28^ab^	1.36	1.02^ab^	1.20	1.73^b^	1.32	1.46	0.94^ab^
	CC	1.07^abc^	1.09^ab^	0.95	1.92^a^	0.94	1.46^b^	0.94	1.22	2.47^a^
	CC + BVg	1.25^ab^	1.42^a^	0.86	1.59^ab^	1.30	0.71^b^	0.95	1.18	1.89^ab^
	CC + ANT	1.28^ab^	1.18^ab^	1.17	1.28^ab^	1.25	0.91^b^	1.36	0.70	0.83^b^
Male	UC	0.93^bc^	1.11^ab^	1.23	0.59^b^	1.97	4.21^a^	1.83	1.14	0.55^b^
	CC	0.80^c^	0.76^b^	0.93	1.65^a^	1.10	1.38^b^	1.23	1.16	1.07^ab^
	CC + BVg	0.82^c^	0.73^b^	0.94	1.81^a^	1.16	1.03^b^	0.99	1.33	1.75^ab^
	CC + ANT	0.85^c^	0.87^ab^	0.97	0.93^ab^	1.42	2.78^b^	1.29	1.06	0.77^ab^
SEM^2^		0.11	0.12	0.13	0.37	0.29	0.50	0.28	0.24	0.33
*P*-Value		0.008	0.003	0.143	0.050	0.452	0.015	0.707	0.573	0.033

^a-b^Values within a column with different letters differ significantly (*P* < 0.05).

^1^Treatment abbreviations: UC: Unchallenged control; CC: Challenged control; BVg: Blend of butyric and valeric glyceride @ 1000 g/t, 500 g/t, 250 g/t in starter, grower, and finisher phases, respectively; ANT: Antibiotic, Zn bacitracin 267 g/t; Salinomycin 500 g/t.

^2^SEM: standard error of means.

### Expression of Mucin Production and Cell Apoptosis Related Genes

The expression of genes related to mucin production and cell apoptosis are shown in Table 8. Dietary treatments significantly affected *MUC2* expression only in male birds (*P* < 0.05). The male birds in the UC group had higher expression of *MUC2* compared to the male birds in the CC groups (*P* < 0.05), whereas the expression of this gene in BVg and ANT groups were not different from any other groups (*P* > 0.05) but showed a shift from CC towards UC. No significant differences in *MUC2* expression were observed in female birds across treatments. Significant sex effects were observed for *CASP8* expression only in BVg fed birds (*P* < 0.05, Table 8), where *CASP8* was upregulated in female birds compared to the male birds but not in other groups (*P* > 0.05).

**Table 8:** Effect of experimental treatment and sex on expression of genes related to mucin production and cell apoptosis in jejunum on d 16.

Sex	Experimental Treatment^1^	*MUC2*	*CASP3*	*CASP8*
Female	UC	1.40^a^	0.95	0.99^ab^
	CC	0.88^ab^	1.11	1.10^ab^
	CC + BVg	1.05^ab^	1.24	1.35^a^
	CC + ANT	1.02^ab^	0.87	1.00^ab^
Male	UC	1.39^a^	1.23	1.00^ab^
	CC	0.82^b^	0.87	0.80^b^
	CC + BVg	0.98^ab^	0.81	0.82^b^
	CC + ANT	0.93^ab^	0.81	0.85^b^
SEM^2^		0.12	0.14	0.09
*P-*value		0.009	0.236	0.009

^a-b^Values within a column with different letters differ significantly (*P* < 0.05).

1 Treatment abbreviations: UC: Unchallenged control; CC: Challenged control; BVg: Blend of butyric and valeric glyceride @ 1000 g/t, 500 g/t, 250 g/t in starter, grower, and finisher phases, respectively; ANT: Antibiotic, Zn bacitracin 267 g/t; Salinomycin 500 g/t.

2 SEM: standard error of means.

### Effect of treatment and sex on genes encoding nutrient transporters

Table 9 shows the effects of dietary treatments and sex on the expression of nutrient transporters. The dietary treatments significantly affected *B^0^AT* expression only in female birds and *b^0,+^AT* in both the male and female birds (*P* < 0.05). The *B^0^AT* expressions were significantly higher in female birds of the UC group compared to the CC group (*P* < 0.05), while the expression levels of BVg and ANT groups showed no differences from CC and UC (*P* > 0.05), showing a shift from CC towards UC group. However, no significant difference was observed in the expression of this gene in male birds across all treatment groups (*P* > 0.05). The *b^0,+^AT* was upregulated in the female birds of the UC group compared to the CC and BVg groups (*P* < 0.05). The expression of this gene in the female bird of ANT group was significantly higher than the CC groups (*P* < 0.05), but not different from the UC and BVg groups (*P* > 0.05). The expression of *b^0,+^AT* in the in female birds of BVg group was not different from those of both the CC and ANT groups (*P* > 0.05), showing a shift towards ANT group. In Addition, *b^0,+^AT* was downregulated in the male bird of the CC and BVg groups compared to those of the UC group (*P* < 0.05). The expression of this gene in the male birds of ANT group was not different from all other groups (*P* > 0.05).

**Table 9:** Experimental treatment and sex effects on nutrient transporter gene expressions in jejunum on d 16.

Sex	Experimental Treatment^1^	*ASCT1*	*B^0^AT*	*b^0,+^AT*	*GLUT2*	*LAT1*	*PepT1*	*PepT2*
Female	UC	1.23^ab^	1.31^a^	1.46^a^	1.33	1.08^ab^	1.87	2.30
	CC	1.26^ab^	0.84^b^	0.77^d^	0.98	1.31^a^	2.31	2.32
	CC + BVg	1.49^a^	1.00^ab^	0.89^bcd^	1.25	1.44^a^	1.90	1.31
	CC + ANT	1.25^ab^	1.20^ab^	1.33^abc^	1.27	1.05^ab^	1.57	2.29
Male	UC	0.85^bc^	1.13^ab^	1.45^ab^	1.37	0.80^b^	0.46	0.95
	CC	0.69^c^	0.90^ab^	0.88^cd^	0.81	0.80^b^	0.66	2.75
	CC + BVg	0.83^bc^	0.88^ab^	0.87^cd^	0.88	0.81^b^	1.79	1.40
	CC + ANT	0.68^c^	1.11^ab^	1.19^abcd^	1.02	0.66^b^	1.53	1.57
SEM		0.10	0.09	0.13	0.15	0.10	0.52	0.54
*P-*value		<0.001	0.009	<0.001	0.086	<0.001	0.381	0.283

^a-c^Values within a column with different letters differ significantly (*P* < 0.05).

1 Treatment abbreviations: UC: Unchallenged control; CC: Challenged control; BVg: Blend of butyric and valeric glyceride @ 1000 g/t, 500 g/t, 250 g/t in starter, grower, and finisher phases, respectively; ANT: Antibiotic, Zn bacitracin 267 g/t; Salinomycin 500 g/t. ^2^SEM: standard error of means.

On the other hand, the sex of the birds had effects on the expression of the *ASCT1* and *LAT1* genes (*P* < 0.05, Table 9). The expression of *ASCT1* was higher in female birds compared to the male birds in the groups CC, BVg and ANT (*P* < 0.05) but not in UC (*P* > 0.05), and *LAT1* in the group of CC and BVg (*P* < 0.05) but not in UC and ANT groups (*P* > 0.05).

## Discussion

Phasing out of in-feed antibiotics due to legislation and consumer demand in poultry production has led to the search for non-antibiotic alternatives to control enteric diseases, including NE in broiler chickens. Organic acids (OAs), particularly SCFA and their esters, salts or coated forms, have shown efficacy in improving performance and controlling NE (Mora et al., 2020; Khan et al., 2022). While the effectiveness of BA is well-documented, the use of VA compounds is relatively new, and their role in modulating gut health and controlling enteric pathogens in any species remains less explored. This study evaluated the impact of BVg on the performance and gut health of broilers, along with the mechanisms involved under a subclinical NE challenge. The findings showed that the BVg supplementation shifted FCR from CC towards the UC group during both the challenge phase and overall study period and EPEF at d35 was similar to that of UC group. BVg supplementation resulted in similar expressions of *IL6, TJP1* and *OCLN* to the UC and ANT groups in both the male and female birds and showed a shift of expression of *MUC2* and *B^0^AT* from CC towards the UC group in male and female birds, respectively. Furthermore, BVg supplementation shifted the oocyst counts from CC towards UC and ANT groups. Therefore, we accept the hypothesis that dietary inclusion of BVg improves gut health and performance in broiler chickens under subclinical NE challenge by modulating genes related to gut health and thus gut microenvironment.

In this study, birds in the CC group exhibited significantly higher FCR compared to the UC group during both the challenge (d 8-19) and overall (d 0-35) periods, indicating the successful induction of sub-clinical NE. Similar performance reductions have been documented in birds with sub-clinical NE (M'Sadeq et al., 2015; Gharib-Naseri et al., 2019; Kumar et al., 2021a). Intestinal damage, increased gut permeability and impaired gut microenvironment are contributors to the poorer performance in these birds as have been reported elsewhere. This was further evidenced by increased oocyst counts, higher serum FITC-d concentration, and altered bacterial population and gene expression related to gut integrity, immunity and nutrient transport in the CC group. Although not significant, a shift of performance parameters, gene expression and oocyst counts were observed in BVg supplemented group from those of CC towards UC and/or ANT groups. The BVg supplemented birds exhibited an EPEF value (409) comparable to that of the unchallenged control group (420), suggesting an overall improvement in flock performance and economic efficiency in broiler production. A higher EPEF value signifies better production results, while an EPEF above 220 is generally considered effective (Van Limbergen et al., 2020). Consistent with our findings, a recent study reported better production efficiency in commercial settings, with EPEF values ranging from 388 in 2021 to 412 in 2023 (Adaszynska-Skwirzynska et al., 2025). BVg supplementation also showed an improvement and shift of AWG and FCR (1.3% and 2.7%, respectively) from CC to the UC group during the challenged period (d 8-19) and a shift of FCR from CC towards UC and ANT groups with 1.7% improvement during overall (d 0-35) study periods compared to the CC group, suggesting beneficial effects in ameliorating NE challenge and, to some extent, improving broiler performance. Similar findings were reported by Gracia et al. (2024), where a combination of BA and VA glyceride with oregano oil improved average daily gain (3%), average daily feed intake (1.5%), and FCR (1.3%) compared to control birds during the overall study period. Another study revealed that both VA and BA glycerol esters were as effective as bacitracin in controlling the growth and efficiency-suppressing negative effects of the necrotic enteritis challenge (Hofacre et al., 2020). Although functionality may vary due to the molecular structure of individual OA, their effects are generally attributed to improved gut health through their antibacterial activity, modulation of gut microflora, stimulation of humoral and cell-mediated immunity, and improvement of intestinal integrity and digestibility, which collectively enhance production performance (Gadde et al., 2017; Bedford and Gong, 2018; Mora et al., 2020; Khan et al., 2022). Gut health directly influences the health status of birds and affects digestion, absorption and metabolism of nutrients, as well as immunity and disease resistance (Kelly and Conway, 2001; Yegani and Korver, 2008). In this study, BVg supplementation shifted the oocyst counts from CC towards UC and ANT groups. Additionally, it elevated the level of *Bifidobacterium* population to the levels similar to UC and ANT groups in male birds. *Bifidobacterium* spp. have been reported to enhance gut health in chicken by promoting a balanced gut microbiota, increasing villus height for better nutrient absorption, and stimulating immune responses, ultimately contributing better feed utilization and overall performance (Naeem and Bourassa, 2025; Idowu et al., 2025). These findings suggest an improved gut microenvironment and support the potentiality of additives to improve performance in broiler chickens under NE challenge. Modulating the expression of genes related to gut integrity, immunity and nutrient transporters is crucial for maintaining a healthy gut and preventing adverse disease impacts. BVg supplementation showed similar *IL6, TJP1* and *OCLN* expression levels to the UC and ANT groups in both the male and female birds and shifted the expression of *MUC2* in males and *B^0^AT* in females from CC towards the UC group. These findings highlight the potentiality of the additive in enhancing nutrient transport, gut immunity, integrity and barrier function. Ultimately, this led to improved protection against pathogen, nutrient digestion and absorption and better overall performance in NE challenge broilers. In addition, butyrate has been shown to be a significant source of energy for enterocytes. It contributes to maintaining gut mucosal health and plays a key role in enhancing the proliferation and differentiation of epithelial cells and intestinal absorption (Canani et al., 2011). On the other hand, VA glycerol esters have been reported to improve performance (3% and 3.3% more in BW and FCR, respectively) under NE challenge in broiler chickens by increasing the length of intestinal villi, suggesting an increased surface area capable of more nutrient absorption and digestion, and by increasing the density of GLP-2 cells in the intestine, which stimulates intestinal growth (Onrust et al., 2018). Therefore, the improved performance in this study could be associated with the synergistic and/or complementary effect of BA and VA glycerides. The combined supplementation likely promotes gut development, mitigates inflammation, stimulates the immune system and modulates gut microbiota, which collectively improve growth, feed intake, and feed conversion efficiency. However, varying responses observed in *Bifidobacterium* colonization and gut-centric gene expression in male and female birds might be attributed to the sex-based physiological, hormonal and microbial factors. Sex has been reported to influence gut immune function, epithelial integrity and nutrient transporters activity, leading to sex-specific modulation of gut-centric gene expression and microbiota composition (Markle et al., 2013; Kers et al, 2018; Kumar et al., 2021b; England et al., 2024). Notably, male chickens have been shown a closer link between their cecal microbiota and glycan metabolism, while females show a link with lipid metabolism (Cui et al., 2021).

Enteric inflammatory diseases, including NE, alter the expression of genes related to immunity, inflammation, mucus secretion, nutrient transporters and tight junctions (Du et al., 2016; Barekatain et al., 2019; Gharib-Naseri et al., 2021). In this study, birds in the CC group exhibited significant downregulation of *IgG, MUC2* and amino acid transporters, *B^0^AT* and *b^0,+^AT* compared to the UC group. Higher oocyst counts and increased serum FITC-d level in the CC group observed in this study suggest substantial intestinal damage, which could impair gut integrity and nutrient uptake and subsequently weaken the immunoglobulin responses. This impairment was further evidenced by higher FCR in the CC group compared to the UC group during the challenge phase and overall study period in the current study. BVg supplementation resulted in an expression shift of *TJP1* and *OCLN* towards the ANT group in both the male and female birds. This indicates its potentiality in improving gut integrity and barrier functions, thereby ensuring proper nutrient absorption, and preventing pathogenic bacterial translocation in broiler chicken. These findings align with a previous study in broilers in which NE-challenged birds fed a glyceride blend (containing mono-, di-, and tri-glycerides of butyric, caprylic, and capric acids) exhibited upregulated *TJP1* expression compared to unchallenged birds (Kumar et al., 2021a). Similarly, studies involving rodents, humans, and in vitro cultures have shown that butyric acid improves intestinal barrier functions by increasing the expression of tight junction proteins (Guilloteau et al., 2010; Wang et al., 2012). In the current study, mRNA expression of *IL6* in the BVg supplemented group showed no difference from the ANT and UC groups in both the male and female birds, with a shift from CC towards the ANT group in the female birds. This indicates that BVg supplementation may help to control local or systemic acute inflammatory responses and mitigate the adverse effects of NE challenge. The blend of butyric and valeric acid glycerides used as additive in this study consist of SCFAs, which have been shown to modulate immune and inflammatory responses through the activation of free fatty acid receptors type 2 and 3 (FFA2 and FFA3 receptors), G protein-coupled receptor 109A (GPR109A), and inhibition of histone deacetylases (HDACs) (Li et al., 2018). Additionally, male birds fed BVg showed a shift in *MUC2* expression level from CC towards the UC, with no significant difference from the ANT group. This indicates the potential of the additive to protect the intestinal mucus layer against NE challenge. Similar findings have been reported previously, where NE-challenged birds supplemented with a monoglyceride blend or a combination of OAs and essential oils exhibited increased *MUC2* expression (Stefanello et al., 2020; Kumar et al., 2021a). Such increased *MUC2* expression is known to have beneficial effects on intestinal health by improving digestion, absorption, nutrient transportation, and protection against pathogens, ultimately enhances broiler performance (Jiang et al., 2013; Duangnumsawang et al., 2021). Furthermore, BVg supplementation shifted the *B^0^AT* and *b^0,+^AT* expression levels from CC towards the UC and ANT groups, respectively in female birds, indicating improved capacity to uptake amino acids into epithelial cells, thereby better performance. Collectively, gene expression findings in the current study suggest that the BVg supplementation may stimulate the expression of some genes related to gut integrity, immunity and amino acid transporters in response to pathogenic infection and improved performance as reflected by an FCR shift towards the UC and ANT groups during both the challenge and overall study periods. This improvement is likely due to enhanced gut integrity and barrier functions, immune stimulation, improved nutrient transportation, and a shift of energy allocation from immune responses to growth.

*Eimeria* oocysts are highly infectious and relatively resistant to unfavorable environments, causing significant damage to the intestinal epithelial cells, which leads to reduced bird performance and increased morbidity and mortality (Jenkins et al., 2019; de Freitas et al., 2023). In the current study, the CC group showed significantly higher oocyst counts compared to the UC groups. Birds receiving the BVg supplementation showed lower oocyst counts, approaching the levels seen in the UC and ANT groups. This reduction could be to a decrease in intracellular pH, where undissociated BVg enters into the coccidian cells, dissociates in the cytoplasm, and causes cell death (Geier et al., 2010). Encapsulated OAs have been shown to enhance anticoccidial drug efficacy, particularly diclazuril in *Eimeria* spp-infected broilers (Nouri, 2022). An *in vitro* and i*n vivo* study by Zurisha et al. (2021) also found that butyric acid inhibits oocyst sporulation, damages oocysts, and also improves chicken performance and parameters related to coccidiosis, such as lesion scores and oocyst counts. In addition to a direct effect on coccidian cells, BVg supplementation improved the expression of genes related to tight junction proteins, mucin production, and immunity, which could prevent the invasion of epithelial cells by parasites and stimulate the self-limiting phenomenon of the disease, resulting in a reduced oocyst count.

## Conclusion

The BVg supplementation showed an overall shift of FCR towards the antibiotic and unchallenged groups, and the EPEF was similar at d35 to that of the UC group. The additive inclusion also shifted the expression of genes encoding gut integrity, immunity and nutrient transporters, which are all in line with the improvement in the FCR. Additionally, BVg supplementation shifted the oocyst count from challenged control towards the unchallenged control and antibiotic groups. Therefore, it can be concluded that BVg supplementation has the potential to improve intestinal health and ameliorate the sub-clinical NE of broilers through improved gut integrity, barrier functions, immunity, nutrient transportation, and gut microenvironment. However, we did not evaluate the dosage effect of BVg at different feed stages and the combinations of different levels of both components. Future studies may include evaluating its dosage effects and varying combinations of BA and VA glycerol esters to optimise the use of BVg under the NE challenge as well as under normal conditions. Further study will also be able to provide more information about the underlying mechanisms.

## Declaration of competing interest

The authors declare the following financial interests/personal relationships which may be considered as potential competing interests: Shu-Biao Wu reports financial support of the research was provided by Perstorp Waspik BV, The Netherlands.

## Appendix Supplementary materials

Image, application 1

## Footnotes

Supplementary material associated with this article can be found, in the online version, at doi:10.1016/j.psj.2025.105441.
