# Supplementary material for: Butyric and valeric glycerides alleviate sub-clinical necrotic enteritis effect on performance and gut health of broiler chickens
Source: Poult Sci. 2025 Jun 13;104(9):105441. doi: 10.1016/j.psj.2025.105441 (PMC12266499; doi:10.1016/j.psj.2025.105441)
Supplement: Supplementary file 1 [file mmc1.docx]

**Table S1. Sequences of primers for RT-qPCR to determine bacterial load in caecal content.**

| **Target group of bacteria** | **Primer sequence (5′→3′)** | **Annealing temperature, ^o^C** | **Reference** |
| --- | --- | --- | --- |
| *Lactobacillus* spp. | F- CAC CGC TAC ACA TGG AG R- AGC AGT AGG GAA TCT TCC A | 63 | Rinttila et al., 2004 |
| *Bifidobacterium* spp. | F- GCG TCC GCT GTG GGC R- CTT CTC CGG CAT GGT GTT G | 63 | Requena et al., 2002 |
| *Bacteroides* spp. | F- GAG AGG AAG GTC CCC CAC R- CGC TAC TTG GCT GGT TCA G | 63 | Layton et al., 2006 |
| *Bacillus* spp. | F- GCA ACG AGC GCA ACC CTT GA R- TCA TCC CCA CCT TCC TCC GGT | 63 | Zhang et al., 2015 |
| *Ruminococcus* spp. | F- GGC GGC YTR CTG GGC TTT R- CCA GGT GGA TWA CTT ATT GTG TTA A | 63 | Ramirez-Farias et al., 2008 |
| Enterobacteriaceae | F- CAT TGA CGT TAC CCG CAG AAG AAG C R- CTC TAC GAG ACT CAA GCT TGC | 63 | Bartosch et al., 2004 |
| Total bacteria | F- CGG YCC AGA CTC CTA CGG G R- TTA CCG CGG CTG CTG GCA C | 63 | Lee et al., 1996 |

**Table S2. Sequence of primers used for qPCR analysis of reference and target genes in jejunum on d16.**

| **Gene^1^** | **Accession No.** | **Sequence** | **Size (bp)** | **T_a_^o^C** | **Reference** |
| --- | --- | --- | --- | --- | --- |
| Reference Gene | | | | | |
| *RPL4* | NM_001007967 | F: TTATGCCATCTGTTCTGCC  R: GCGATTCCTCATCTTACCCT | 235 | 60 | Yang et al., 2013 |
| *β-ACT* | AB495656.1 | F-CTCTGACTGACCGCGTTACTCC  R- CCATACCAACCATCACACCCTG | 175 | 60 | Kheravii et al., 2018 |
| Genes related to cell apoptosis | | | | | |
| *CASP3* | NM_204725.1 | F-TGGTGGAGGTGGAGGAGC  R- GTTTCTCTGTATCTTGAAGCACCA | 110 | 62 | Gharib-Naseri et al., 2020 |
| *CASP8* | NM_204592.4 | F- GGAGCTGCTCTATCGGATCAAT  R- AGCAGATACCTGAACGGAGACAC | 126 | 60 | This study |
| Immune genes | | | | | |
| *IgA* | S40610.1 | F: GTCACCGTCACCTGGACACCA  R: ACCGATGGTCTCCTTCACATC | 192 | 64 | Lammers et al., 2010 |
| *IgG* | X07174.1 | F: ATCACGTCAAGGGATGCCCG  R: ACCAGGCACCTCAGTTTGG | 118 | 60 | Lammers et al., 2010 |
| *IgM* | X01613.1 | F: GCATCAGCGTCACCGAAAGC  R: TCCGCACTCCATCCTCTTGC | 98 | 60 | Lammers et al., 2010 |
| *IFN-y* | Y07922 | F: AGCTGACGGTGGTGGACCTATTATT  R: GGCTTTGCGCTGGATTC | 259 | 60 | Li et al., 2018 |
| *IL-2* | NM_204153.1 | F: TCTGGGACCACTGTATGCTCT  R: ACACCAGTGGGAAACAGTATCA | 256 | 60 | Liu et al., 2018 |
| *IL-6* | NM_204628.1 | F: GATCCGGCAGATGGTGATAA  R: AGGATGAGGTGCATGGTGAT | 126 | 60 | Song et al., 2021 |
| Mucin production gene | | | | | |
| *MUC2* | XM 001234581.3 | F: CCCTGGAAGTAGAGGTGACTG  R: TGACAAGCCATTGAAGGACA | 143 | 60 | Fan et al., 2015 |
| Integrity genes | | | | | |
| *OCLN* | NM_205128.1 | F: ACGGCAGCACCTACCTCAA  R: GGGCGAAGAAGCAGATGAG | 123 | 60 | Du et al., 2016 |
| *TJP1* | XM_413773.4 | F: GGATGTTTATTTGGGCGGC  R: GTCACCGTGTGTTGTTCCCAT | 187 | 60 | Zanu et al., 2020 |
| *JAM2* | XM_046907882.1 | F-AGACAGGAACAGGCAGTGCTAG  R-ATCCAATCCCATTTGAGGCTAC | 135 | 60 | Kumar et al., 2021 |
| Transporter genes | | | | | |
| *ASCT1* | XM 001232899.4 | F-TTGGCCGGGAAGGAGAAG  R: AGACCATAGTTGCCTCATTGAATG | 63 | 60 | Paris and Wong, 2013 |
| *b^0,+^AT* | NM_001199133.1 | F: CAGTAGTGAATTCTCTGAGTGTGAAGCT  R: GCAATGATTGCCACAACTACCA | 88 | 60 | Gilbert et al., 2007 |
| *B^0^AT* | XM_419056.5 | F: GTGTTTGGAACCCTAAATACGAGG  R: TAGCATAGACCCAGCCAGGA | 72 | 60 | Kheravii et al., 2018 |
| *GLUT2* | NM_207178.1 | F: TGATCGTGGCACTGATGGTT  R: CCACCAGGAAGACGGAGATA | 171 | 60 | Kheravii et al., 2018 |
| *LAT1* | KT876067.1 | F: GATTGCAACGGGTGATGTGA  R: CCCCACACCCACTTTTGTTT | 70 | 60 | Gilbert et al., 2007 |
| *PepT1* | AY029615.1 | F: TACGCATACTGTCACCATCA  R: TCCTGAGAACGGACTGTAAT | 205 | 60 | Guo et al., 2014 |
| *PepT2* | NM_001319028.1 | F: TGACTGGGCATCGGAACAA  R: ACCCGTGTCACCATTTTAACCT | 63 | 60 | Paris and Wong, 2013 |

^1^Gene names: *RPL4:* Ribosomal protein L4, *β-ACT:* Actin β, *CASP3:* Caspase-3, *CASP8:* Caspase-8, *IFN-y:* interferon gamma, *IgA:* immunoglobulin A, *IgG:* immunoglobulin G, *IgM:* immunoglobulin M, *IL-2:* interleukin 2, *IL-6:* interleukin 6, *MUC2:* Mucin 2, *OCLN:* Occludin, *TJP1 (ZO-1):* Tight junction protein 1 (Zonula occludens-1), *JAM2:* Junctional Adhesion Molecule 2, *ASCT1:* Alanine, serine, cysteine, and threonine transporter, *B^0^AT:* Solute carrier family 6, member 19 (SLC6A19), *b^0,+^AT*: Solute carrier family 7, member 9 (SLC7A9), GLUT2: Glucose transporter 2, *LAT1:* L-Type amino acid transporter 1, *PepT1:* Peptide transporter-1, *PepT2:* Peptide transporter-2.

**Table S3. Effect of BVg on intestinal lesion score of broilers on d 16 under sub-clinical necrotic enteritis challenge**

| **Treatment^1^** | **Duodenum** | | **Jejunum** | |
| --- | --- | --- | --- | --- |
|  | Male | Female | Male | Female |
| UC | 0.22 | 0.11 | 0.06 | 0.17 |
| CC | 0.35 | 0.20 | 0.70 | 0.45 |
| CC + BVg | 0.61 | 0.13 | 0.50 | 0.39 |
| CC + ANT | 0.22 | 0.17 | 0.28 | 0.39 |
| SEM^2^ | 0.18 | 0.15 | 0.19 | 0.11 |
| *P*-value | 0.407 | 0.891 | 0.107 | 0.275 |

^1^Treatment abbreviations: UC: Unchallenged control; CC: Challenged control; BVg: Blend of butyric and valeric glyceride @ 1000 g/t, 500 g/t, 250 g/t in starter, grower, and finisher phases, respectively; ANT: Antibiotic, Zn bacitracin 267 g/t; Salinomycin 500 g/t.

^2^SEM: standard error of means.

**Table S4. Effects of BVg on the SCFA concentrations (μmol/g) in caecal digesta of broilers**.

| **Treatments** | **SCFA (µmol/g)** | | | | | | | | |
| --- | --- | --- | --- | --- | --- | --- | --- | --- | --- |
|  | **Formic acid** | **Acetic acid** | **Propionic acid** | **Isobutyrate** | **Butyrate** | **Isovalerate** | **Valerate** | **Lactate** | **Succinate** |
| UC | 0.61 | 42.6 | 1.72 | 0.40 | 10.3 | 0.13 | 0.40 | 0.03 | 13.8 |
| CC | 0.38 | 48.0 | 4.60 | 0.51 | 16.1 | 0.17 | 0.63 | 0.01 | 9.46 |
| CC + BVg | 0.60 | 60.8 | 6.78 | 0.54 | 14.5 | 0.18 | 0.69 | 1.24 | 7.89 |
| CC + ANT | 0.60 | 54.7 | 2.65 | 0.38 | 13.7 | 0.07 | 0.43 | 0.24 | 17.5 |
| SEM | 0.17 | 6.26 | 1.39 | 0.07 | 1.74 | 0.03 | 0.48 | 0.11 | 3.51 |
| *P-*value | 0.667 | 0.208 | 0.071 | 0.207 | 0.132 | 0.075 | 0.155 | 0.231 | 0.230 |

SCFA: short chain fatty acids

^1^Treatment abbreviations: UC: Unchallenged control; CC: Challenged control; BVg: Blend of butyric and valeric glyceride @ 1000 g/t, 500 g/t, 250 g/t in starter, grower, and finisher phases, respectively; ANT: Antibiotic, Zn bacitracin 267 g/t; Salinomycin 500 g/t.

^2^SEM: standard error of means.

**REFERENCES**

Bartosch, S., Fite, A., Macfarlane, G.T., McMurdo, M.E., 2004. Characterization of bacterial communities in feces from healthy elderly volunteers and hospitalized elderly patients by using real-time PCR and effects of antibiotic treatment on the fecal microbiota. Appl. Environ. Microbiol. 70, 3575–3581.

Du, E, W Wang, L Gan, Z Li, S Guo, and Y Guo. 2016. Effects of thymol and carvacrol supplementation on intestinal integrity and immune responses of broiler chickens challenged with *clostridium perfringens*. J. Anim. Sci. Biotechnol. 7, 1-10.

Fan, X., Liu, S., Liu, G., Zhao, J., Jiao, H., Wang, X., Song, Z., Lin, H., 2015. Vitamin a deficiency impairs mucin expression and suppresses the mucosal immune function of the respiratory tract in chicks. PLoS. One 10, e0139131.

Gharib-Naseri, K, JC de Paula Dorigam, K Doranalli, S Kheravii, RA Swick, M Choct, and S-B Wu. 2020. Modulations of genes related to gut integrity, apoptosis, and immunity underlie the beneficial effects of *Bacillus amyloliquefaciens* cect 5940 in broilers fed diets with different protein levels in a necrotic enteritis challenge model. J. Anim. Sci. Biotechnol. 11:1-13.

Gilbert, E., Li, H., Emmerson, D., Webb Jr, K., Wong, E., 2007. Developmental regulation of nutrient transporter and enzyme mrna abundance in the small intestine of broilers. Poult. Sci. 86, 1739–1753.

Guo, S., Liu, D., Zhao, X., Li, C., Guo, Y., 2014. Xylanase supplementation of a wheat-based diet improved nutrient digestion and mrna expression of intestinal nutrient transporters in broiler chickens infected with *Clostridium perfringens*. Poult. Sci. 93, 94–103.

Kheravii, S.K., Swick, R.A., Choct, M., Wu, S.B., 2018. Upregulation of genes encoding digestive enzymes and nutrient transporters in the digestive system of broiler chickens by dietary supplementation of fiber and inclusion of coarse particle size corn. BMC. Genomics. 19, 1–14.

Kumar, A., Toghyani, M., Kheravii, S.K., Pineda, L., Han, Y., Swick, R.A., Wu, S.B., 2021b. Potential of blended organic acids to improve performance and health of broilers infected with necrotic enteritis. Anim. Nutr. 7, 440–449.

Lammers, A., Wieland, W.H., Kruijt, L., Jansma, A., Straetemans, T., Schots, A., den Hartog, G., Parmentier, H.K., 2010. Successive immunoglobulin and cytokine expression in the small intestine of juvenile chicken. Dev. Comp. Immunol. 34, 1254–1262.

Layton, A., McKay, L., Williams, D., Garrett, V., Gentry, R., Sayler, G., 2006. Development of bacteroides 16s rrna gene taqman-based real-time pcr assays for estimation of total, human, and bovine fecal pollution in water. Appl. Environ. Microbiol. 72, 4214–4224.

Lee, D.H., Zo, Y.G., Kim, S.J., 1996. Nonradioactive method to study genetic profiles of natural bacterial communities by pcr-single-strand-conformation polymorphism. Appl. Environ. Microbiol. 62, 3112–3120.

Li, Z., Wang, W., Liu, D., Guo, Y., 2018. Effects of lactobacillus acidophilus on the growth performance and intestinal health of broilers challenged with clostridium perfringens. J. Anim. Sci. Biotechnol. 9, 1-10.

Liu, N., Wang, J.Q., Liu, Z.Y., Chen, Y.K., Wang, J.P., 2018. Tetramethylpyrazine attenuates necrotic enteritis by reducing gut oxidative stress, inflammation, opportunistic bacteria and endotoxins in broilers. Eur. Poult. Sci. 82, 233.

Paris, N., Wong, E., 2013. Expression of digestive enzymes and nutrient transporters in the intestine of *Eimeria maxima*-infected chickens. Poult. Sci. 92, 1331–1335.

Ramirez-Farias, C., Slezak, K., Fuller, Z., Duncan, A., Holtrop, G., Louis, P., 2008. Effect of inulin on the human gut microbiota: stimulation of *bifidobacterium adolescentis* and *faecalibacterium prausnitzii*. Br. J. Nutr. 101, 541–550.

Requena, T., Burton, J., Matsuki, T., Munro, K., Simon, M.A., Tanaka, R., Watanabe, K., Tannock, G.W., 2002. Identification, detection, and enumeration of human *bifidobacterium* species by pcr targeting the transaldolase gene. Appl. Environ. Microbiol. 68, 2420–2427.

Rinttila, T., Kassinen, A., Malinen, E., Krogius, L., Palva, A., 2004. Development of an extensive set of 16s rdna-targeted primers for quantification of pathogenic and indigenous bacteria in faecal samples by real-time pcr. J. Appl. Microbiol. 97, 1166–1177.

Song, B., Tang, D., Yan, S., Fan, H., Li, G., Shahid, M.S., Mahmood, T., Guo, Y., 2021. Effects of age on immune function in broiler chickens. J. Anim. Sci. Biotechnol. 12, 1–12.

Yang, F., Lei, X., Rodriguez-Palacios, A., Tang, C., Yue, H., 2013. Selection of reference genes for quantitative real-time pcr analysis in chicken embryo fibroblasts infected with avian leukosis virus subgroup j. BMC. Res. Notes. 6, 1–5.

Yang, F., Lei, X., Rodriguez-Palacios, A., Tang, C., Yue, H., 2013. Selection of reference genes for quantitative real-time pcr analysis in chicken embryo fibroblasts infected with avian leukosis virus subgroup j. BMC. Res. Notes. 6, 1–5.

Zanu, H., Keerqin, C., Kheravii, S., Morgan, N., Wu, S., Bedford, M., Swick, R., 2020. Influence of meat and bone meal, phytase, and antibiotics on broiler chickens challenged with subclinical necrotic enteritis: 1. Growth performance, intestinal ph, apparent ileal digestibility, cecal microbiota, and tibial mineralization. Poult. Sci. 99, 1540–1550.

Zhang, Y., Chen, D., Yu, B., He, J., Yu, J., Mao, X., Wang, J., Luo, J., Huang, Z., Cheng, G., 2015. Spray-dried chicken plasma improves intestinal digestive function and regulates intestinal selected microflora in weaning piglets. J. Anim. Sci. 93, 2967–2976.
